# Performance evaluation of plazomicin susceptibility testing of Enterobacterales on VITEK 2 and VITEK 2 Compact Systems

**DOI:** 10.1128/jcm.00449-25

**Published:** 2025-08-01

**Authors:** Edith Csiki-Fejer, Maria Traczewski, Gary W. Procop, Thomas E. Davis, Meredith Hackel, Gilles Zambardi

**Affiliations:** 1 bioMérieux, Inc.Hazelwood, Missouri, USA; 2 Clinical Microbiology Institute, Inc.Wilsonville, Oregon, USA; 3 Cleveland Clinic 2569 Cleveland, Ohio, USA; 4 Indiana University School of Medicine Indianapolis 12250 Indianapolis, Indiana, USA; 5 IHMA Inc. 167022 Schaumburg, Illinois, USA; 6 bioMérieux La-Balme-les-Grottes, France; Cleveland Clinic, Cleveland, Ohio, USA

**Keywords:** antimicrobial susceptibility testing, VITEK 2 AST-GN plazomicin, Enterobacterales

## Abstract

The objective of the present study was to evaluate the VITEK 2 antimicrobial susceptibility test (AST) for Gram-negative (GN) plazomicin performance using the VITEK 2 and VITEK 2 Compact Systems in clinical settings and to demonstrate its equivalence to the Clinical and Laboratory Standards Institute (CLSI) broth microdilution (BMD) technique. The clinical study conducted at four external sites followed the requirements detailed in the US Food and Drug Administration (FDA) and International Standards Organization (ISO) guidance documents and included clinical, challenge, quality, and reproducibility studies. In this multisite study, a total of 979 Enterobacterales isolates were tested. The VITEK 2 card minimum inhibitory concentration (MIC) results were compared with the reference BMD MIC results. The performance following ISO criteria indicated Essential Agreement (EA) = 97.8% and showed that VITEK 2 plazomicin MIC results for Enterobacterales tend to be in exact agreement when compared with the CLSI BMD reference method, except for *Escherichia coli,* when MICs tend to be in exact agreement or at least one doubling dilution lower. The analysis was also performed following the FDA criteria using the breakpoints defined by the FDA: ≤2 susceptible (S), 4 intermediate (I), and ≥8 resistant (R) for Enterobacterales. The analysis showed the following performance: EA = 98.7%, category agreement (CA) = 99.4%, and major errors (ME) = 0.1%, with no very major errors (VME) present. There are three species, *Klebsiella pneumoniae*, *Escherichia coli,* and *Serratia marcescens,* for which the trend is ≥ 30% and therefore addressed as a note in the US label. The plazomicin test met the ISO and FDA criteria of ≥95% reproducibility and ≥95% quality control (QC) results within acceptable ranges for QC organisms.

**IMPORTANCE:**

The VITEK 2 AST-GN plazomicin test is a new, automated alternative to the BMD reference method for determining minimum inhibitory concentrations (MIC) of Enterobacterales, expanding the range of automatic AST testing.

## INTRODUCTION

Plazomicin (ZEMDRI, Cipla Therapeutics) is a new-generation semisynthetic aminoglycoside (sisomicin analog) (1) antibiotic, administered as an intravenous injection, recommended for the treatment of adults (18 years of age or older) with complicated urinary tract infections (cUTIs), including pyelonephritis, and limited or no alternative treatment options (2). It was developed to target multidrug-resistant Enterobacterales by employing chemical modifications to sisomicin (3) that protect against multiple aminoglycoside-modifying enzymes (AME) (3, 4). Its activity in pathogens resistant to older aminoglycosides (amikacin, gentamicin, and tobramycin) is due to its chemical structure that defends plazomicin from enzymes (transferase–acetyltransferases, nucleotidyltransferases, and phosphotransferases) that otherwise may lead to drug inactivation by degradation (3–5).

In addition to receiving the US Food and Drug Administration (FDA) approval in 2018, plazomicin received approval from the Central Drugs Standard Control Organization (CDSCO) (6) to be launched on the pharmaceutical market in India in February 2024. It is not available in Europe, as the marketing company withdrew its application in 2020 (7).

Plazomicin has been shown to be active against Gram-negative bacilli within the Enterobacterales, including *Escherichia coli, Klebsiella pneumoniae*, *Proteus mirabilis*, *Enterobacter cloacae*, *Citrobacter freundii*, *Citrobacter koseri*, *Klebsiella aerogenes*, *Klebsiella oxytoca*, *Morganella morganii*, *Proteus vulgaris*, *Providencia stuartii,* and *Serratia marcescens* as listed in the pharmaceutical label (2).

Plazomicin is a bactericidal aminoglycoside that binds to the bacterial 30S ribosomal subunit, inhibiting protein synthesis. Due to its mechanism of action, which is different from that of beta-lactam antibiotics, it has broad activity against isolates producing β-lactamases, including extended-spectrum beta-lactamase, (TEM, SHV, CTX-M, and AmpC), serine carbapenemases (KPC-2, KPC-3), and oxacillinase (OXA-48). As mentioned, it is active against most AMEs (3) and metallo-beta-lactamase-producing isolates that do not co-express 16S rRNA ribosomal methyltransferase (2). Enterobacterales with 16S rRNA methyltransferases are uncommon in the United States (8, 9) and were mainly isolated in East Asia and, sometimes, co-expressed with NDM (4). Nevertheless, when present, ribosomal methyltransferases protect the ribosomes and prevent the binding of virtually all aminoglycoside molecules, including plazomicin, causing high resistance levels (8).

Plazomicin may have reduced activity against Enterobacterales that overexpress certain efflux pumps (e.g. acrAB-tolC) or lower expression of porins (e.g. ompF or ompK36) (2).

Plazomicin is administered daily (or less frequently, depending on the underlying renal function) as a single dose of intravenous injection. Besides being practical, this way of administration greatly improves the efficacy and the safety profile of plazomicin when compared with older aminoglycosides and presents less frequent and decreased side effects such as nephrotoxicity, ototoxicity, headache, nausea, vomiting, diarrhea, hypertension, and hypotension. It is very important to mention that Plazomicin is less ototoxic (4), and the renal damage (nephrotoxicity) caused by plazomicin is reversible (4, 8).

We investigated the performance of the automated VITEK 2 AST-GN plazomicin *in vitro* quantitative test for determining the minimum inhibitory concentration (MIC) for plazomicin using clinical isolates collected by the external sites.

In this paper, we summarize the MIC VITEK 2 card results compared with the MIC results obtained using the Clinical and Laboratory Standards Institute (CLSI) broth microdilution (BMD) reference method (10). The results obtained for clinical isolates, the challenge set, and the reproducibility set, accompanied by quality control (QC) testing, were analyzed following the FDA (11) and ISO (12) requirements.

## MATERIALS AND METHODS

The VITEK 2 AST-GN plazomicin test performance was evaluated concurrently, in the same study as the VITEK 2 AST-GN omadacycline performance (13). The clinical samples collected and tested at the four external sites (IUSM, Indianapolis, IN; IHMA Schaumburg, IL; CMI Wilsonville, OR; CC Cleveland, OH) were bacterial microorganisms that originated from clinical human residual specimen cultures. The clinical isolates included contemporary (isolated less than 6 months and, if frozen, minimally subcultured, to maintain the original culture with minimal changes) and stock isolates with no time constraints, selected to complement the contemporary isolates. All 869 clinical frozen organisms were subcultured on 2 consecutive days (from frozen isolates, to isolate a pure strain from a single species of bacteria) onto trypticase soy agar with 5% sheep blood (TSAB; Thermo Fisher Scientific Remel Products, Lenexa, KS) and incubated at 35 ± 2°C for 18–24 h before testing. Pure culture colonies were suspended in 3 mL of sterile aqueous 0.45% NaCl to obtain the standard inoculum (0.5–0.63 McFarland suspension, turbidity measured employing DensiCHEK Plus instrument). The same standard inoculum was used to inoculate the VITEK 2 AST-GN cards of both VITEK 2 and VITEK 2 Compact instruments (14, 15) and the BMD panels within 30 min of preparation. VITEK 2 and VITEK 2 Compact instruments are part of the VITEK 2 Systems product line and use the same cards. VITEK 2 instrument can be used to perform testing in two different dilution workflows, automatic and manual, whereas VITEK 2 Compact operates in manual dilution mode only. The clinical isolates were tested on the VITEK 2 instrument with the automatic dilution option only. Cards were automatically filled, sealed, and loaded into the VITEK 2 instrument for incubation and reading. The instrument monitors the growth of each well in the card up to 18 h for GN cards. At the completion of the incubation cycle, MIC values are automatically determined for each antimicrobial contained on the card.

In addition to the clinical isolates, a set of 110 challenge isolates with on-scale MIC results was developed at bioMérieux and tested in the same manner at CMI, CC, and STLCA. Each challenge isolate was tested once using the same initial 0.5–0.63 McFarland suspension using VITEK 2 automatic and manual dilution modes, as well as VITEK 2 Compact, which functions in manual dilution mode only. Challenge BMD reference results from all participating sites were used to calculate the voted standard. The voted standard was the mode of results for the three sites. This voted standard was compared to the challenge VITEK 2 card results from the CMI site.

The reproducibility, or the precision of the test, was assessed using a set of 10 isolates supplied to the sites by bioMérieux. The reproducibility set was tested with the VITEK 2 AST-GN card at three sites (IHMA, IUSM, and STLCA), and the results were compared within the sites and between the sites. The reproducibility set was tested on the VITEK 2 System with the automatic dilution option as well as the manual dilution option and on the VITEK 2 Compact instrument. Each isolate was tested for 3 days, each day in triplicate using separate bacterial inoculum suspensions, for each dilution mode mentioned above. The card MIC results were compared with the card result mode for each organism. The mode was calculated using only on-scale results. Best-case and worst-case reproducibility results for each site individually and all sites combined were calculated.

Quality control strains, *Escherichia coli* ATCC 25922 and *Pseudomonas aeruginosa* ATCC 27853, listed in the CLSI M100 standard (16), were tested on each day of comparative testing with both the VITEK 2 AST-GN card and the BMD reference method. The ancillary QC organism *S. aureus* ATCC 29213 was tested with the BMD reference method only to perform further QC of the BMD panels. The QC isolates were subcultured twice onto TSAB and incubated at 35 + 2°C for 18–24 hours before testing.

Three different lots of VITEK 2 AST-GN Investigational Use Only (IUO) cards, including plazomicin covering the range from ≤0.5 µg/mL to ≥16 µg/mL, were used during the study. The plazomicin concentration in the cards was 2 µg/mL, 4 µg/mL, and 8 µg/mL. The MIC values are determined by monitoring the isolate growth within each of the 64 µm-wells of the card (13, 14). Every 15 min throughout the incubation cycle (less than 16 h), visible light transmittance readings of each well determine organism growth by the amount of light that is prevented from passing through the well. After the incubation period, the MIC values for each antimicrobial on the card are displayed as an automatically generated report (17). The VITEK 2 MIC results were compared with the BMD panel MIC results following the CLSI procedure (10, 16).

The BMD panels were poured at bioMérieux and contained plazomicin ranging in concentration from 0.062 µg/mL to 256 µg/mL. The BMD panels were prepared with fresh media at the time of panel preparation and subsequently frozen for later use (16). The BMD panels' MIC values were read after the panels were incubated at 35 ± 2°C in ambient air for 16–20 h.

Each external site was granted permission to perform the testing according to the protocols and obtained institutional review board (IRB) approval for exemption (waiver), as no human subjects were included in this trial.

### Data analysis

The performance of the VITEK 2 AST-GN Plazomicin was evaluated according to the FDA (11) and ISO (12) guidance documents for combined clinical and challenge (automatic dilution), challenge VITEK 2 manual dilution, and VITEK 2 Compact manual dilution results.

The results were analyzed using FDA breakpoints for Enterobacterales (18), and the performance was evaluated based on essential agreement (EA), category agreement (CA), minor error (mE), major error (ME), and very major error (VME) rates.

EA is met when the result of the BMD reference method and that of the VITEK 2 AST-GN plazomicin test are within one doubling dilution. CA is met when the interpretation of the BMD reference method result (susceptible, intermediate, and resistant) is the same as the interpretation of the VITEK 2 plazomicin test. Acceptable values for EA and CA are ≥ 90%. The mE rate is defined as the percentage of isolates that show intermediate MIC values by the BMD reference method but are rendered susceptible or resistant by VITEK 2 methods, or alternatively show resistant or susceptible MIC values by the BMD reference method but are intermediate by VITEK 2 methods. The ME rate is defined as the percentage of isolates that are susceptible by the BMD reference method but are resistant by the VITEK 2 methods (acceptable if ≤ 3.0%). The VME rate is defined as the percentage of isolates resistant by the BMD reference method but susceptible by the VITEK 2 method. The acceptable VME rate is ≤ 2% (19).

A trending analysis was also conducted for the combined clinical and challenge (automatic) data.

Species for which the difference between the percentage of isolates with higher or lower test MIC values was ≥ 30% with a statistically significant confidence interval (CI) were considered to have evidence of trending and must be addressed in the labeling.

When applying the 2021 ISO criteria (12), performance is acceptable when EA ≥ 90% and the bias is within 30% (−30% ≤ bias ≤ 30%). Discrepancy (error) resolution was performed for EA errors as well as concordant clinical samples. Error resolution was performed for five isolates by retesting the discrepant isolate in triplicate on the VITEK 2 card and the reference method using separate bacterial inoculum suspensions.

## RESULTS 

### VITEK 2 AST-GN plazomicin

#### ISO performance - overall (clinical and challenge combined)

**Fig 1:**
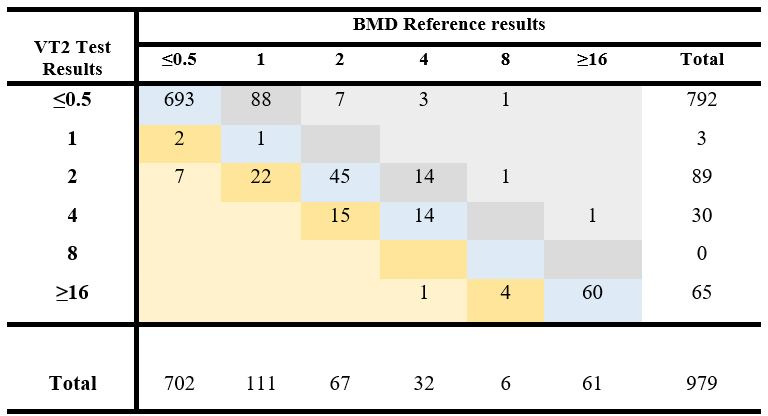
Distribution of MIC values of VITEK 2 AST-GN Plazomicin and the BMD reference method-–Enterobacterales ISO performance–EA and bias calculation. Clinical and challenge isolates (979) were tested with VITEK 2 AST-GN Plazomicin and the BMD reference method. The number of isolates with exact MIC agreement for the Plazomicin test and BMD reference method is shown on a blue background (813). The numbers of isolates with test results greater than the reference are shown on a gold background. The isolates with test results less than the reference are shown on a gray background.

A total of 979 clinical and challenge isolates were tested by the VITEK 2 AST-GN plazomicin and BMD reference methods, and the correlation between the two methods was evaluated according to ISO 20776-2, 2021 guidance. When calculating EA, the range of MICs for the test and reference method to be compared must be the same. In our case, the BMD MIC range was wider (≤0.06 to ≥ 256 µg/mL) than the test MIC (≤0.5 to ≥ 16 µg/mL). To align with the requirements, the reference values from ≤0.06 to ≤0.5 µg/mL were combined. Similarly, the reference MICs at the upper end of the scale from ≥16 µg/mL to ≥256 µg/mL were combined to result in a symmetric diagram. Figure 1 shows the distribution of MIC values of the plazomicin test (VITEK two auto-dilution mode) and the BMD reference method of 869 clinical isolates and 110 challenge isolates. In addition, Table 1 summarizes the EA for the clinical isolates and the challenge isolates tested by all three modes (auto, manual, and compact manual).

**TABLE 1:** VITEK 2 and VITEK 2 Compact Plazomicin ISO performance: Essential Agreement by the panel of isolates^*a*^^,^^*b*^^,*c*^

Type of isolates	MIC doubling dilution difference distribution (Test MIC - Reference MIC)							Essential agreement
	≤ −3	−2	−1	0	+1	+2	≥ +3	
Overall: Clinical, Challenge (Auto-Dilution)	**4**	**9**	**102**	**813**	**43**	**8**	**0**	**958/979** (**97.8%**)
Clinical (Auto-dilution)	3	8	94	726	30	8	0	850/869 (97.8%)
Challenge (Auto-Dilution)	1	1	8	87	13	0	0	108/110 (98.2%)
Challenge (Manual Dilution)	1	2	6	90	11	0	0	107/110 (97.3%)
Challenge (VITEK2 Compact)	1	1	9	88	11	0	0	108/110 (98.2%)

^*a*^ The shaded cells represent the MIC values in the EA.

^*b*^ The EA values meet the acceptance criteria ( EA ≥ 90%).

^*c*^ The bold values refer to the combined (overall) performance of the clinical and challenge sets determined in auto-dilution mode.

The shaded cells represent the MIC values in EA. The EA for overall clinical and challenge (auto-dilution) was 97.8% (958/979). Clinical set EA 97.8% (850/869), and challenge EA values (auto 98.2%, manual 97.3%, and Compact manual 98.2%) all exceeded 90% and therefore meet the acceptance criteria in each situation.

In Table 2, we present the overall EA for clinical and challenge isolates tested with an auto-dilution mode by organism group. All organism groups meet the acceptance criteria.

**TABLE 2:** VITEK 2 Plazomicin ISO performance–Essential Agreement by organism group–auto-dilution^*a*^^,^^*b*^

Organism group	MIC doubling dilution difference distribution (Test MIC - Reference MIC)							Essential agreement
	≤ −3	−2	−1	0	+1	+2	≥ +3	
*Enterobacter / Klebsiella*	0	0	3	417	11	4	0	431/435 (99.1%)
*Escherichia*	0	3	62	259	4	0	0	325/328 (99.1%)
Other Enterobacterales	1	0	1	46	0	0	0	47/48 (97.9%)
*Proteus / Providencia / Morganella*	3	6	28	68	28	4	0	124/137 (90.5%)
*Serratia*	0	0	8	23	0	0	0	31/31 (100.0%)

^*a*^ The shaded cells represent those in the EA.

^*b*^ The EA values meet the acceptance criteria.

Furthermore, the EA distribution within each group is presented in Tables S1 to S5 for the following species: the *Enterobacter/Klebsiella group* (435 isolates) consisting of *E. cloacae* (33) *E. cloacae* complex (3), *K. aerogenes* (30), *K. oxytoca* (32), *K. pneumoniae* (337), *K. pneumoniae* ssp *pneumoniae* (3); the *Escherichia* group consisting entirely of *E. coli* (328); other Enterobacterales, such as *C. freundii* (14) and *C. koseri* (34); and the *Proteus/Providencia/Morganella* group (137) comprised of *M. morganii* (16), *P. mirabilis* (84), *P. vulgaris* (17), *P. stuartii* (20), and *S. marcescens* (31) tested isolates. EA values meet the acceptance criteria at the species level in each situation except in the case of *M. morganii,* where the EA was below 90%. Out of the 16 isolates tested, only 14 were in EA, resulting in an EA of 87.5%. (Table S4).

The bias of the test was calculated for the 979 clinical and challenge isolates tested on VITEK 2 auto-dilution mode and the BMD reference method using the same overall distribution frequency of MIC values between the VITEK 2 test and the BMD as used for EA calculation (Fig. 1). In this context, the bias is the evaluation of the VITEK 2 test results to determine whether the results that differ from the reference method are predominantly in one direction.

The number of isolates with exact MIC agreement for the plazomicin test and BMD reference method is shown on a blue background (813).

The number of isolates with test results greater than the reference is shown on a gold background. There are 51 test results greater than the reference. The number of isolates that have reference values in the range from the lowest to next to the highest MIC, or (≤0.5–8) is 918. Therefore, the percentage of results greater than the reference is (51/918)*100 = 5.6%.

The isolates with test results less than the reference are shown on a gray background. There are 115 test results less than the reference. The number of isolates that have reference values in the range from the next to the lowest MIC to the highest, or (1 to ≥16), is 277. The percentage of test MIC results less than the reference was (115/277)*100 = 41.5%. The overall bias was −35.9%. Values for bias that are contained in the interval, −30% ≤ bias ≤ 30%, are considered acceptable. In this case, the bias was < −30%; therefore, there is a tendency to produce test results that are less than the reference. To address this bias, additional analysis of values below the reference was performed at the group level for *Enterobacter/Klebsiella*, *Escherichia,* other Enterobacterales*, Proteus/Providencia/Morganella,* and *Serratia*. It was found that MIC test results for *Escherichia* were below reference MIC (65/88; 73.9%), at a rate significantly greater than 0%. (Fig. S1). As a consequence, a bias statement must be applied to the *Escherichia* group in the label.

(The additional analysis of bias below the reference was incomplete for the other Enterobacterales and *Serratia* since there were fewer than 15 isolates in the denominator, and at least 25 on-scale isolates should be available when evaluating the bias. When the number of on-scale isolates is less than 25, the EA becomes the only performance metric).

#### FDA performance: clinical isolates

In Table 3, we present the performance of the clinical and challenge set evaluated using the VITEK 2 auto-dilution mode and compared with the BMD reference panel results. The results were analyzed using FDA breakpoints for Enterobacterales (18, 20) ( ≤2 (S), 4 (I), ≥8 (R)).

**TABLE 3:** VITEK 2 Plazomicin FDA performance^*a*^

Org. source	Organism	#	EA #	EA %	CA #	CA%	S #	I #	R #	VME #(%)	ME #(%)	mE #(%)
Clinical	*C. freundii*	14	14	100.0	14	100.0	14	0	0	0(0.0)	0(0.0)	1(6.7)
	*C. koseri*	34	33	97.1	33	97.1	33	1	0	0(0.0)	0(0.0)	0(0.0)
	*E. cloacae*	27	27	100	27	100	27	0	0	0(0.0)	0(0.0)	0(0.0)
	*E. cloacae* complex	3	3	100.0	3	100.0	3	0	0	0(0.0)	0(0.0)	0(0.0)
	*E. coli*	299	296	99.0	299	100.0	299	0	0	0(0.0)	0(0.0)	0(0.0)
	*K. aerogenes*	30	27	90.0	30	100.0	30	0	0	0(0.0)	0(0.0)	0(0.0)
	*K. oxytoca*	30	28	93.3	30	100.0	30	0	0	0(0.0)	0(0.0)	0(0.0)
	*K. pneumoniae*	300	299	99.7	299	99.7	299	0	1	0(0.0)	1(0.3)	0(0.0)
	*P. vulgaris*	16	15	93.8	15	93.8	15	1	0	0(0.0)	0(0.0)	1(6.3)
	*S. marcescens*	30	30	100	30	100	30	0	0	0(0.0)	0(0.0)	0(0.0)
	Clinical Total	783	772	98.6	780	99.6	781	2	1	0(0.0)	1(0.1)	2(0.3)
Challenge automatic dilution	*E. cloacae*	6	6	100.0	6	100.0	0	0	6	0(0.0)	0(0.0)	0(0.0)
	*E. coli*	29	29	100.0	28	99.6	13	2	14	0(0.0)	0(0.0)	1(3.2)
	*K. oxytoca*	2	2	100.0	2	100.0	2	0	0	0(0.0)	0(0.0)	0(0.0)
	*K. pneumoniae ssp. ozaenae*	1	1	100.0	1	100.0	0	0	1	0(0.0)	0(0.0)	0(0.0)
	*K. pneumoniae ssp pneumoniae*	1	1	100.0	1	100.0	0	0	1	0(0.0)	0(0.0)	0(0.0)
	*K. pneumoniae*	35	35	100.0	34	97.1	1	0	34	0(0.0)	0(0.0)	1(2.9)
	*S. marcescens*	1	1	100	1	100	1	0	0	0(0.0)	0(0.0)	0(0.0)
	Challenge Total	75	75	100.0	73	97.3	17	2	56	0(0.0)	0(0.0)	1(1.3)
Combined	Overall	858	847	98.7	853	99.4	797	4	57	0(0.0)	1(0.1)	4(0.5)

^*a*^ EA (Essential Agreement); CA (Category Agreement); S (Susceptible); I (Intermediate); R (Resistant); VME (Very Major Error); ME (Major Error); mE (minor Error).

The clinical set included *C. freundii (14*), *C. koseri (34*), *E. cloacae (27), E. cloacae* complex (*3), E. coli (299), K. aerogenes (30*), *K. oxytoca (30*), *K. pneumoniae (300*), *P. vulgaris (16*), and *S. marcescens (30*). Of these isolates, 62.1% (486/783) were contemporary isolates (collected and tested at the sites within 6 months, minimally subcultured) and 37.9% (297/783) were stock isolates.

For the clinical organisms evaluated, the overall EA (98.6%) and CA (99.6%) were excellent. The EA and CA were acceptable ( ≥90%) at each species level as shown in Table 3. No VME was observed, only one ME and 2 mE were registered.

#### FDA performance: challenge isolates

A total of 75 challenge isolates, including *E. cloacae* (6), *E. coli* (29), *K. oxytoca* (2)*, K. pneumoniae* (37), and *S. marcescens* (1) were tested at three sites on VITEK 2 and VITEK Compact.

The challenge set was selected to include a high number of resistant isolates, comprising 74.7% (56/75) of the total set. The performances of the challenge isolates were evaluated on VITEK 2 using auto and manual dilution modes and on VITEK 2 Compact (manual) and were compared with the BMD reference panel results.

For VITEK 2 using auto-dilution mode, the EA for challenge isolates was 100% (75/75), and the CA was 97.3% (73/75). There were no ME or VME, and only 2 mE were observed. All individual species had EA and CA higher than 90% (Table 3).

Performance results for challenge isolates using VITEK 2 manual dilution mode were EA 98.7% (74/75) and CA 97.3% (73/75). There were no ME or VME. All individual species had EA and CA higher than 90%.

VITEK 2 Compact manual dilution mode provided an EA for challenge isolates of 100% (75/75) and CA of 98.7% (74/75). There were no ME or VME. All individual species had EA and CA higher than 90%.

#### FDA performance: overall (clinical and challenge combined)

**Fig 2:**
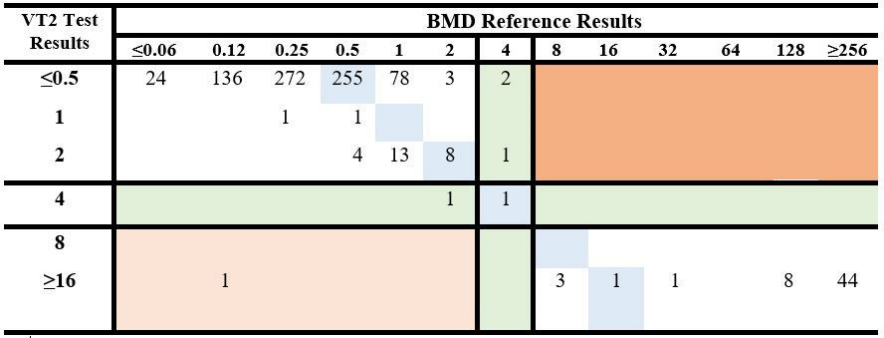
Distribution of MIC Values of VITEK 2 AST-GN Plazomicin and the BMD reference method–Enterobacterales FDA performance. Clinical and challenge auto-dilution isolates (858) were tested with VITEK 2 AST-GN Plazomicin and the BMD reference method. Plazomicin FDA breakpoints: ≤2 (S), 4 (I), ≥8 (R). Test range ≤0.5 µg/mL - ≥ 16 µg/mL and complete BMD range ≤0.06 µg/mL - ≥ 256 µg/mL. The number of isolates with the same MIC for the plazomicin test and BMD reference method is indicated on a blue background. Out of 858 tests, 847 results were in EA (98.7%). The number of isolates constituting ME is shown on a light orange background (1) while mEs are on a green background (4). There were no VME (dark orange) registered.

Figure 2 shows the distribution frequency of the test MIC and BMD MIC reference method values for the 858 Enterobacterales isolates included in the FDA validation set.

The left column shows the VITEK 2 (VT2) test range ≤0.5 - ≥ 16 µg/mL, while the BMD two-fold serial dilution concentration range is shown horizontally from ≤0.06 to ≥256 µg/mL. Results were analyzed using FDA breakpoints ≤ 2 (S), 4 (I), ≥8 (R).

Based on BMD MIC values, 92.9% (797/858) of isolates were susceptible, 0.5% (4/858) were intermediate, and 6.6% (57/858) were resistant.

Resistance to plazomicin was not common for isolates collected at the clinical sites; only one resistant *K. pneumoniae* isolate was enrolled. The resistant isolates tested (*E. cloacae (6), E. coli (14), K. pneumoniae subsp. ozaenae (1), K. pneumoniae subsp. pneumoniae (1), K. pneumoniae (34*)) were part of the challenge set and selected for resistance testing.

Despite our efforts, the ability of the AST-GN card to detect resistance for six species tested (*C. fruendii, C. koseri, K. aerogenes, K. oxytoca, P. vulgaris,* and *S. marcescens*) is unknown because resistant strains were not available at the time of comparative testing.

The EA, CA, and error rates were calculated for all Enterobacterales and individual species (Fig. 2; Table 3).

The overall performance following FDA criteria was 98.7% (847/858) for EA and 99.4% (853/858) for CA. There was one ME (1/797; 0.1%), four mEs (4/858; 0.5%), and no VMEs observed. (When testing *Morganella morganii, Proteus mirabilis,* and *Providencia stuartii*, clinical and automatic challenge results did not meet the expected EA and CA FDA performance criteria. Consequently, those were excluded from the claim and final performance results.)

#### Trending analysis

A trending analysis was conducted for the combined clinical and challenge data obtained from the VITEK 2 auto-dilution method. We considered MIC values that are on-scale and are one or more doubling dilutions lower or higher than the reference method. (MIC values that are off-scale for both the reference and device are not considered in the trending analysis.) Therefore, evaluable results for trending were not available for *E. cloacae, E. cloacae* complex, *K. pneumoniae subsp. ozaenae*, and *K. pneumoniae subsp. pneumoniae*.

The trending results summarized in Table 4 show the percent difference between higher and lower MIC values and the 95% CI (CI). There are three species *K. pneumoniae*, *Escherichia coli,* and *Serratia marcescens,* for which the trending is significant (≥ 30%) and therefore addressed as a note in the US labeling. The MIC values tended to be in exact agreement or at least one doubling dilution higher when testing *Klebsiella pneumoniae* and in exact agreement or at least one doubling dilution lower when testing *Escherichia coli* and *Serratia marcescens* compared to the CLSI reference BMD method.

**TABLE 4:** Plazomicin trending analysis: Clinical and challenge isolates - Auto-Dilution

Organism	Total evaluable for trending	≥1 Dil. Lower # (%)	Exact # (%)	≥1 Dil. higher # (%)	Percent difference (95% CI)	Trending noted
*C. freundii*	1	1 (100.00%)	0 (0.00%)	0 (0.00%)	−100.00% (−100.00, 12.21)	No
*C. koseri*	2	1 (50.00%)	1 (50.00%)	0 (0.00%)	−50.00% (−90.55, 27.26)	No
*K. aerogenes*	4	1 (25.00%)	0 (0.00%)	3 (75.00%)	50.00% (−13.55, 78.91)	No
*E. coli*	74	65 (87.84%)	5 (6.76%)	4 (5.41%)	−82.43% (-88.95,-70.32)	Yes
*K. oxytoca*	5	1 (20.00%)	0 (0.00%)	4 (80.00%)	60.00% (−0.03, 83.16)	No
*K.pneumoniae*	10	1 (10.00%)	0 (0.00%)	9 (90.00%)	80.00% (36.99, 91.61)	Yes
*Proteus vulgaris*	12	6 (50.00%)	2 (16.67%)	4 (33.33%)	−16.67% (−48.09, 20.32)	No
*S.marcescens*	9	8 (88.89%)	1 (11.11%)	0 (0.00%)	−88.89% (-98.01,-44.80)	Yes
TOTAL	117	84 (71.79%)	9 (7.69%)	24 (20.51%)	−51.28% (-60.99,-39.29)	Yes

#### Time of call

The card incubation time was recorded for all 980 challenge and clinical isolates. The incubation cycle for the VITEK 2 system is expected to be less than 16 hours. For the tested clinical isolates, the mean incubation time was 7.47 hours, with a standard deviation of 1.57. The incubation/testing time exceeded 16 hours only six times (6/869, 0.7%) for four *K. pneumoniae* isolates and one *K. aerogenes* clinical isolate. Statistical data for all Enterobacterales included in the trial and for all three testing modes for challenge isolates are presented in Table S8.

#### Reproducibility

The reproducibility set consisted of 10 isolates (*E. cloacae* (3), and *K. pneumoniae* subsp. *pneumoniae* (7)). Each isolate was tested in triplicate for each dilution mode (VITEK 2 automatic and manual, and VITEK 2 Compact manual dilution modes) using separate inocula for three separate days at the two external (IUSM, IHMA), and one internal trial site (STLCA). Each isolate had 27 results for each method. A total of 270 data points were collected. The mode of MIC values was determined for each isolate, and the reproducibility was calculated based on the number of MIC values that fell within ±1 doubling dilution of the mode MIC value. The majority of data points were within ±1 doubling dilution agreement as compared to the mode MIC. The differences between the card results and the card results mode for all 270 tests are presented in Table S6.

The best-case scenario assumes that the MIC values for off-scale organisms are within one doubling dilution of the mode, while the worst-case scenario assumes that the MIC values for off-scale organisms are greater than one doubling dilution from the mode (Tables S6 and S7).

The VITEK two plazomicin reproducibility for automatic dilution was 97.04% (262/270) in the best case and 97.04% (262/270) in the worst case. The VITEK two plazomicin reproducibility for manual dilution was 95.6% (258/2700) for the best case and 95.6% (258/270) for the worst case. The VITEK 2 Compact plazomicin reproducibility for manual dilution was 97.4% (262/2700) - best-case and 97.4% (262/270) - worst-case.

In every instance, reproducibility tests met the ≥95% FDA and ISO criteria and the new FDA requirement for worst-case ≥89% (21).

#### Quality control

As recommended in the CLSI M100 standard (16), two QC strains were tested during each day of comparative testing with the VITEK 2 (auto and manual dilution modes), VITEK 2 Compact (manual), and the BMD reference method containing plazomicin. The testing was performed a minimum of 20 times at each testing site for *Escherichia coli* ATCC 25922 (range 0.25 µg/mL to 2 µg/mL) and *Pseudomonas aeruginosa* ATCC 27853 (range 1 µg/mL to 4 µg/mL). In addition, one Gram-positive *S. aureus* ATCC 29213 (range 0.25 µg/mL to 2 µg/mL), an ancillary QC organism, was tested by the BMD reference method only to perform further QC of the BMD panels.

The results of this testing are summarized in Tables S9 and S10.

In every case, VITEK 2 results for each dilution mode were within the acceptable range ≥ 95% of the time and met the FDA and ISO requirements (11, 12).

## DISCUSSION

Plazomicin is a semisynthetic aminoglycoside derived from the naturally occurring sisomicin (22), one of the few antimicrobials developed in recent years that has the potential to be an effective alternative to the treatment of infections caused by multidrug-resistant Enterobacterales, including carbapenem-resistant, extended-spectrum β-lactamase-producing Enterobacterale*s* and organisms resistant to other aminoglycosides. It was designed to minimize vulnerability to aminoglycoside-modifying enzymes (AMEs) by employing deoxo sugars (23, 24) and proved greater activity compared with amikacin, gentamicin, and tobramycin against difficult-to-treat Enterobacterales (25). It is an important option for treating MBL-associated cUTIs or can be used compassionately if available (26). It is inactive against *A. baumannii* and *S. maltophilia*, while its activity against *P. aeruginosa* is variable (26).

Plazomicin is already available for antimicrobial susceptibility testing in the form of ETEST Plazomicin strips (27, 28), HardyDisk, Liofilchem MTS, and ThermoFisher Scientific Sensititre MIC Plates.

The new VITEK 2 AST-GN plazomicin test is the first automated AST system, which was developed as an alternative to the BMD for susceptibility testing, to determine the MIC in Enterobacterales. The testing range covers concentrations from ≤ 0.5 µg/mL to ≥ 16 µg/mL.

Data collected during the trial were analyzed in two separate ways, following both the ISO and the FDA requirements, as presented in the Methods and Results sections.

The ISO performance was determined as EA and Bias for the following organisms: *C. freundii*, *C. koseri*, *E. cloacae, E. cloacae* complex*, E. coli, K. aerogenes*, *K. oxytoca*, *K. pneumoniae*, *P. vulgaris*, *M. morganii, P. mirabilis*, *P. stuartii,* and *S. marcescens*.

The EA, defined as the card MIC result within ±1 two-fold dilution from the MIC of the reference method for overall clinical and challenge (auto-dilution) was 97.8% (958/979). EA values meet the acceptance criteria at the group level and the species level except for *M. morganii* (87.5%). Nevertheless, there was no statistically significant difference between species, and the combined value for the *Proteus/Providencia/Morganella* group was 90.5% with a 90% CI (85.3, 94.3), therefore acceptable.

Bias along the entire testing range was calculated by comparing the percentage of results greater than the reference to the percentage of results less than the reference and was found to be −35.9%, a tendency toward test results that are less than the reference method, due to the *Escherichia* group. Therefore, a bias statement will be added to the ISO label specifying that VITEK 2 plazomicin MIC results for *Escherichia* tend to be in exact agreement or at least one doubling dilution lower when compared to the CLSI BMD reference method.

The overall combined clinical and challenge FDA performance was EA = 98.7% and CA = 99.4%, with one ME (0.1%) and no VME registered. A breakdown by species and the MIC correlation are presented in Fig. 2 and Table 3 and show excellent agreement between VITEK 2 and BMD reference MIC values and provide a clear description of the device’s accuracy and capacity to give a correct MIC value of the Plazomicin test.

When testing *M. morganii, P. mirabilis, and P. stuartii*, clinical and automatic challenge results did not meet the expected FDA performance criteria. As a consequence, those were excluded from the claim and final performance results. In addition, to inform customers, an alternative method limitation was proposed to be added to the FDA labeling for plazomicin and the above-mentioned organisms (17).

As a result of the Trend Analysis, there are three species for which a trending note is required. VITEK 2 plazomicin MIC values tended to be in exact agreement or at least one doubling dilution higher when testing *K. pneumoniae* and in exact agreement or at least one doubling dilution lower when testing *Escherichia coli* and *Serratia marcescens* compared to the CLSI reference BMD method (17).

In the United States, the VITEK 2 AST-GN plazomicin test is validated for AST testing of GN bacilli and is intended to be used with VITEK 2 systems to determine the susceptibility of the following microorganisms: *E. coli, K. pneumoniae*, *E. cloacae*, *C. freundii*, *C. koseri*, *K. aerogenes*, *K. oxytoca*, *P. vulgaris,* and *S. marcescens*.

The VITEK 2 test met the ISO and FDA criteria of ≥ 95% reproducibility as follows: best-case reproducibility for automatic dilution was 97.0%, for manual dilution was 95.6%, and for Compact manual dilution was 97.0%, proving the high precision of the test (Table S7).

Two QC organisms were tested each day of testing according to CLSI recommendations. The BMD dilution range (≤ 0.0625 µg/mL - ≥ 128 µg/mL) covered the full expected result range of *E. coli* ATCC 25922 and *P. aeruginosa* ATCC 27853 (Table S9). However, the VITEK 2 Plazomicin card (≤ 0.5 µg/mL - ≥ 16 µg/mL) resulted in off-scale MIC values for *E. coli* ATCC 25922. An *E. coli* ATCC 25922 MIC value of ≤ 0.5 µg/mL (with VITEK 2) was considered an indication that the quality control test results were acceptable. To address the potential off-scale QC results when testing *E. coli* ATCC 25922, a footnote will be added to the label to indicate that the device does not include the full CLSI/FDA recommended dilution range for QC testing (17).

Throughout the study, the QC results were within acceptable ranges (≥ 95%) for all QC organisms tested (Tables S9 and S10).

The incubation time for clinical isolates exceeded 16 hours only six times (0.7%, 6/869). The mean time of call was 7.47 hours, much shorter than the typical BMD overnight results. These results demonstrate that the plazomicin VITEK 2 test leads to faster AST results than the BMD method, consequently significantly reducing diagnostic time.

Fifty-seven resistant isolates were tested, proving that VITEK 2 AST-GN Plazomicin can accurately detect resistance. However, the ability of the card to detect resistance with *C. freundii, C. koseri, K. aerogenes, K. oxytoca, P. vulgaris,* and *S. marcescens* is unknown because resistant strains were not available at the time of testing.

In conclusion, the results of the presented multicenter clinical study demonstrated a reliable correlation between the newly developed VITEK 2 AST-GN Plazomicin test for Enterobacterales and the CLSI BMD reference technique. Our results validate the use of the plazomicin AST test (cards) in conjunction with VITEK 2 and VITEK 2 Compact Systems using 9.04 software or higher as an automated IVD tool to determine MICs in Enterobacterales. The VITEK 2 AST-GN plazomicin (card range from ≤0.5 µg/mL to ≥16 µg/mL) received FDA clearance in February 2023.

## EDITOR'S NOTE

**Ed. Note:** A potential conflict of interest was identified after acceptance of this paper, and the editor in chief performed an additional final review of the paper.

## SUPPLEMENTAL MATERIAL

Supplemental tables and figure jcm.00449-25-s0001.docx Tables S1 to S10 and Fig. S1.

ASM does not own the copyrights to Supplemental Material that may be linked to, or accessed through, an article. The authors have granted ASM a non-exclusive, world-wide license to publish the Supplemental Material files. Please contact the corresponding author directly for reuse.
