# Supplementary material for: Performance evaluation of plazomicin susceptibility testing of Enterobacterales on VITEK 2 and VITEK 2 Compact Systems
Source: J Clin Microbiol. 2025 Aug 1;63(9):e00449-25. doi: 10.1128/jcm.00449-25 (PMC12421896; doi:10.1128/jcm.00449-25)
Supplement: Supplemental tables and figure — Tables S1 to S10 and Fig. S1. [file jcm.00449-25-s0001.docx]

**Supplemental Material**

**Performance Evaluation of Plazomicin Susceptibility Testing of Enterobacterales on VITEK^®^ 2** **and VITEK^®^ 2 Compact Systems**

**Edith Csiki-Fejer****^1^, Maria Traczewski^2^, Gary W. Procop^3^, Thomas E. Davis^4^, Meredith Hackel^5^, Gilles Zambardi^6^**

^1^bioMérieux, Inc., Hazelwood, MO, USA

^2^Clinical Microbiology Institute, Inc., Wilsonville, OR, USA

^3^Cleveland Clinic, Cleveland, OH, USA

^4^Indiana University School of Medicine Indianapolis, IN, USA

^5^IHMA Inc., Schaumburg, IL, USA

^6^bioMérieux, La-Balme-les-Grottes, France

*Corresponding author: Edith Csiki-Fejer, edith.csiki-fejer@biomerieux.com

Keywords: Antimicrobial Susceptibility Testing, VITEK^®^ 2 AST-GN Plazomicin, Enterobacterales

Running header: **VITEK^®^ 2** **Plazomicin** **Performance Evaluation**

## Table S1 Essential Agreement (by Species - *Enterobacter/Klebsiella*) - Clinical, Challenge (Auto Dilution) Difference Distribution (Test MIC - Reference MIC)

| ***Enterobacter/Klebsiella*** | **MIC Doubling Dilution Difference Distribution** | | | | | | | | **Essential Agreement** |
| --- | --- | --- | --- | --- | --- | --- | --- | --- | --- |
|  | **≤ -3** | **-2** | **-1** | **0** | | **+1** | **+2** | **≥ +3** |  |
| Combined | 0 | 0 | 3 | 417 | | 11 | 4 | 0 | 431/435 (99.1%) |
| *Enterobacter cloacae* pooled(1) | 0 | 0 | 0 | 36 | | 0 | 0 | 0 | 36/36 (100.0%) |
| *Enterobacter cloacae* | 0 | 0 | 0 | 33 | | 0 | 0 | 0 | 33/33 (100.0%) |
| *Enterobacter cloacae* complex | 0 | 0 | 0 | 3 | | 0 | 0 | 0 | 3/3 (100.0%) |
| *Klebsiella aerogenes* | 0 | 0 | 1 | 26 | | 0 | 3 | 0 | 27/30 (90.0%) |
| *Klebsiella oxytoca* | 0 | 0 | 1 | | 27 | 3 | 1 | 0 | 31/32 (96.9%) |
| *Klebsiella pneumoniae* pooled(2) | 0 | 0 | 1 | 328 | | 8 | 0 | 0 | 337/337 (100.0%) |
| *Klebsiella pneumoniae* | 0 | 0 | 1 | 325 | | 8 | 0 | 0 | 334/334 (100.0%) |
| *Klebsiella pneumoniae ssp pneumoniae* | 0 | 0 | 0 | 3 | | 0 | 0 | 0 | 3/3 (100.0%) |

^(1)^*Enterobacter cloacae* pooled group consists of *Enterobacter cloacae* and *Enterobacter cloacae* complex.

^(2)^*Klebsiella pneumoniae* pooled group consists of *Klebsiella pneumoniae* and *Klebsiella pneumoniae ssp pneumoniae*

## Table S2 EA (by Species - *Escherichia*) - Clinical, Challenge (Auto Dilution) Difference Distribution (Test MIC - Reference MIC)

| ***Escherichia*** | **MIC Doubling Dilution Difference Distribution** | | | | | | | **Essential Agreement** |
| --- | --- | --- | --- | --- | --- | --- | --- | --- |
|  | **≤ -3** | **-2** | **-1** | **0** | **+1** | **+2** | **≥ +3** |  |
| Combined | 0 | 3 | 62 | 259 | 4 | 0 | 0 | 325/328 (99.1%) |
| *Escherichia coli* | 0 | 3 | 62 | 259 | 4 | 0 | 0 | 325/328 (99.1%) |

## Table S3 EA (by Species - Other Enterobacterales) - Clinical, Challenge (Auto Dilution) Difference Distribution (Test MIC - Reference MIC)

| **Other *Enterobacterales*** | **MIC Doubling Dilution Difference Distribution** | | | | | | | **Essential Agreement** |
| --- | --- | --- | --- | --- | --- | --- | --- | --- |
|  | **≤ -3** | **-2** | **-1** | **0** | **+1** | **+2** | **≥ +3** |  |
| Combined | 1 | 0 | 1 | 46 | 0 | 0 | 0 | 47/48 (97.9%) |
| *Citrobacter freundii* | 0 | 0 | 1 | 13 | 0 | 0 | 0 | 14/14 (100.0%) |
| *Citrobacter koseri* | 1 | 0 | 0 | 33 | 0 | 0 | 0 | 33/34 (97.1%) |

## Table S4 EA (by Species - *Proteus / Providencia / Morganella*) - Clinical, Challenge (Auto Dilution) Difference Distribution (Test MIC - Reference MIC)

| ***Proteus / Providencia / Morganella*** | **MIC Doubling Dilution Difference Distribution** | | | | | | | **Essential Agreement** |
| --- | --- | --- | --- | --- | --- | --- | --- | --- |
|  | **≤ -3** | **-2** | **-1** | **0** | **+1** | **+2** | **≥ +3** |  |
| Combined | 3 | 6 | 28 | 68 | 28 | 4 | 0 | 124/137 (90.5%) |
| *Morganella morganii* | 1 | 1 | 11 | 3 | 0 | 0 | 0 | 14/16 (87.5%) |
| *Proteus mirabilis* | 1 | 5 | 9 | 49 | 18 | 2 | 0 | 76/84 (90.5%) |
| *Proteus vulgaris* | 1 | 0 | 6 | 6 | 4 | 0 | 0 | 16/17 (94.1%) |
| *Providencia stuartii* | 0 | 0 | 2 | 10 | 6 | 2 | 0 | 18/20 (90.0%) |

## Table S5 EA (by Species - *Serratia*) - Clinical, Challenge (Auto Dilution) Difference Distribution (Test MIC - Reference MIC)

| ***Serratia*** | **MIC Doubling Dilution Difference Distribution** | | | | | | | **Essential Agreement** |
| --- | --- | --- | --- | --- | --- | --- | --- | --- |
|  | **≤ -3** | **-2** | **-1** | **0** | **+1** | **+2** | **≥ +3** |  |
| Combined | 0 | 0 | 8 | 23 | 0 | 0 | 0 | 31/31 (100.0%) |
| *Serratia marcescens* | 0 | 0 | 8 | 23 | 0 | 0 | 0 | 31/31 (100.0%) |

## Table S6. Reproducibility performance of VITEK 2 AST-GN Plazomicin. The card result mode is calculated for on-scale results only. Card range ≤ 0.5 - ≥ 16 μg/mL

| **Organism** | **Method** | **Dilution Differences btv. Card Result and Card Result Mode** | | | | | | | **Card Result Mode (µg/mL)** |
| --- | --- | --- | --- | --- | --- | --- | --- | --- | --- |
|  |  | **Off-scale** | **-2** | **-1** | **0** | **+1** | **+2** | **Off-scale** |  |
| ***Enterobacter cloacae*** | VT2 Auto-Dilution | 2 |  |  | 25 |  |  |  | 2 |
|  | VT2 Manual Dilution | 6 |  | 1 | 19 | 1 |  |  | 2 |
|  | Compact Manual Dilution | 5 |  | 3 | 19 |  |  |  | 2 |
| ***Klebsiella pneumoniae* ssp*. pneumoniae*** | VT2 Auto-Dilution |  |  | 2 | 25 |  |  |  | 4 |
|  | VT2 Manual Dilution |  |  | 1 | 26 |  |  |  | 4 |
|  | Compact Manual Dilution |  |  |  | 27 |  |  |  | 4 |
| ***Enterobacter cloacae*** | VT2 Auto-Dilution |  |  |  | 24 | 3 |  |  | 2 |
|  | VT2 Manual Dilution |  |  |  | 27 |  |  |  | 2 |
|  | Compact Manual Dilution |  |  |  | 26 | 1 |  |  | 2 |
| ***Enterobacter cloacae*** | VT2 Auto-Dilution | 1 |  |  | 22 | 4 |  |  | 2 |
|  | VT2 Manual Dilution | 2 |  |  | 24 |  | 1 |  | 2 |
|  | Compact Manual Dilution | 1 |  | 1 | 25 |  |  |  | 2 |
| ***Klebsiella pneumoniae* ssp. *pneumoniae*** | VT2 Auto-Dilution |  |  | 1 | 26 |  |  |  | 2 |
|  | VT2 Manual Dilution |  |  |  | 27 |  |  |  | 2 |
|  | Compact Manual Dilution |  |  |  | 27 |  |  |  | 2 |
| ***Klebsiella pneumoniae* ssp*. pneumoniae*** | VT2 Auto-Dilution |  |  |  | 17 | 5 |  | 5 | 4 |
|  | VT2 Manual Dilution |  |  |  | 17 | 7 |  | 3 | 4 |
|  | Compact Manual Dilution |  |  |  | 18 | 7 |  | 2 | 4 |
| ***Klebsiella pneumoniae* ssp*. pneumoniae*** | VT2 Auto-Dilution |  |  | 4 | 23 |  |  |  | 4 |
|  | VT2 Manual Dilution |  |  | 12 | 15 |  |  |  | 4 |
|  | Compact Manual Dilution |  |  | 13 | 14 |  |  |  | 4 |
| ***Klebsiella pneumoniae* ssp*. pneumoniae*** | VT2 Auto-Dilution |  |  | 6 | 21 |  |  |  | 4 |
|  | VT2 Manual Dilution |  |  | 7 | 20 |  |  |  | 4 |
|  | Compact Manual Dilution |  |  | 7 | 20 |  |  |  | 4 |
| ***Klebsiella pneumoniae* ssp*. pneumoniae*** | VT2 Auto-Dilution |  |  |  | 16 | 11 |  |  | 2 |
|  | VT2 Manual Dilution |  |  |  | 19 | 8 |  |  | 2 |
|  | Compact Manual Dilution |  |  |  | 22 | 5 |  |  | 2 |
| ***Klebsiella pneumoniae ssp. pneumoniae*** | VT2 Auto-Dilution |  |  |  | 27 |  |  |  | 2 |
|  | VT2 Manual Dilution |  |  |  | 27 |  |  |  | 2 |
|  | Compact Manual Dilution |  |  |  | 27 |  |  |  | 2 |
| **Total** | VT2 Auto-Dilution | 3 | 0 | 13 | 226 | 23 | 0 | 5 |  |
|  | Best-Case Combined Reproducibility 262/270 =97.04% | | | | | | | | |
| **Total** | VT2 Manual Dilution | 8 | 0 | 21 | 221 | 16 | 1 | 3 |  |
|  | Best-Case Combined Reproducibility 258/270 =95.56% | | | | | | | | |
| **Total** | Compact Manual Dilution | 6 | 0 | 24 | 225 | 13 | 0 | 2 |  |
|  | Best-Case Combined Reproducibility 262/270 =97.04% | | | | | | | | |

## Table S7: Reproducibility Performance for VITEK 2 AST-GN Plazomicin VITEK 2 Auto-Dilution, Manual Dilution, and VITEK 2 Compact Manual Dilution.

| Reproducibility | VITEK 2 | | VITEK 2 Compact |
| --- | --- | --- | --- |
|  | Auto Dilution | Manual Dilution | Manual Dilution |
| Best-case | 97.0% | 95.6% | 97.0% |
| Worst-case | 97.0% | 95.6% | 97.0% |

## Table S8. VITEK 2 Time of Call – by Sample Type

| Sample Type | Dilution | Total Samples | Mean | Std. Dev. | Min | Max | # Calls  > 16 Hours | | %Calls >16 Hours |
| --- | --- | --- | --- | --- | --- | --- | --- | --- | --- |
| Challenge | Automatic | 110 | 8.03 | 1.73 | 5.73 | 13.13 | 0 | 0.0 | |
| Challenge | Manual | 110 | 8.17 | 1.95 | 5.83 | 16.02 | 1 | 0.9 | |
| Challenge | Compact | 110 | 8.56 | 1.98 | 6.02 | 16.78 | 1 | 0.9 | |
| Clinical | Automatic | 869 | 7.47 | 1.57 | 5.55 | 18.00 | 6 | 0.7 | |

## Table S9. VITEK 2 System Quality Control Summary

| QC Strain | CLSI QC Range µg/mL | VITEK2 Card QC Range µg/mL | No (%) VITEK 2  Results Within Range | | | % Reference Results Within Range |
| --- | --- | --- | --- | --- | --- | --- |
|  |  |  | Auto | Manual | Compact |  |
| *ATCC^®^ 25922 E. coli* | 0.25– 2 | ≤0.5– 2 | 203/204  (99.5) | 98/98  (100.0) | 100/100  (100) | 100.0 |
| *ATCC^®^2785 P. aeruginosa* | 1 - 4 | 1 – 4 | (202/203) 99.5 | (97/97) 100.0 | (98/98) 100.0 | 100.0 |

## Table S10 Ancillary Quality Control Summary:

| QC Strain | CLSI  QC Range  µg/mL | Total Tested | Number Reference Results Within Range | % Reference Results Within Range |
| --- | --- | --- | --- | --- |
| ATCC*^®^* 29213 *S. aureus* | 0.25-2 | 207 | 207 | 100 |

## Figure S1 - Distribution of MIC values of VITEK 2 AST-GN Plazomicin and the BMD reference method -– *Escherichia* ISO Performance - Bias

The number of isolates with exact MIC agreement for the Plazomicin test and BMD reference method are shown on a blue background.

The number of isolates with test results greater than the reference are shown on a gold background. The isolates with test results less than reference are shown on a grey background

| **VT2 Test results** | **Reference (BMD) results** | | | | | | |
| --- | --- | --- | --- | --- | --- | --- | --- |
|  | **≤0.5** | **1** | **2** | **4** | **8** | **≥16** | **Total** |
| **≤0.5** | 240 | 61 | 3 |  |  |  | 304 |
| **1** |  |  |  |  |  |  | 0 |
| **2** |  | 4 | 4 | 1 |  |  | 6 |
| **4** |  |  |  | 1 |  |  | 1 |
| **8** |  |  |  |  |  |  | 0 |
| **≥16** |  |  |  |  |  | 14 | 14 |
|  |  |  |  |  |  |  |  |
| **Total** | 240 | 65 | 7 | 2 | 0 | 14 | 328 |
